# Supplementary material for: High-throughput feedback-enabled optogenetic stimulation and spectroscopy in microwell plates
Source: Commun Biol. 2023 Nov 24;6:1192. doi: 10.1038/s42003-023-05532-4 (PMC10673842; doi:10.1038/s42003-023-05532-4)
Supplement: Supplementary file 2 — Supplementary Information [file 42003_2023_5532_MOESM2_ESM.pdf]

Supplementary Figures

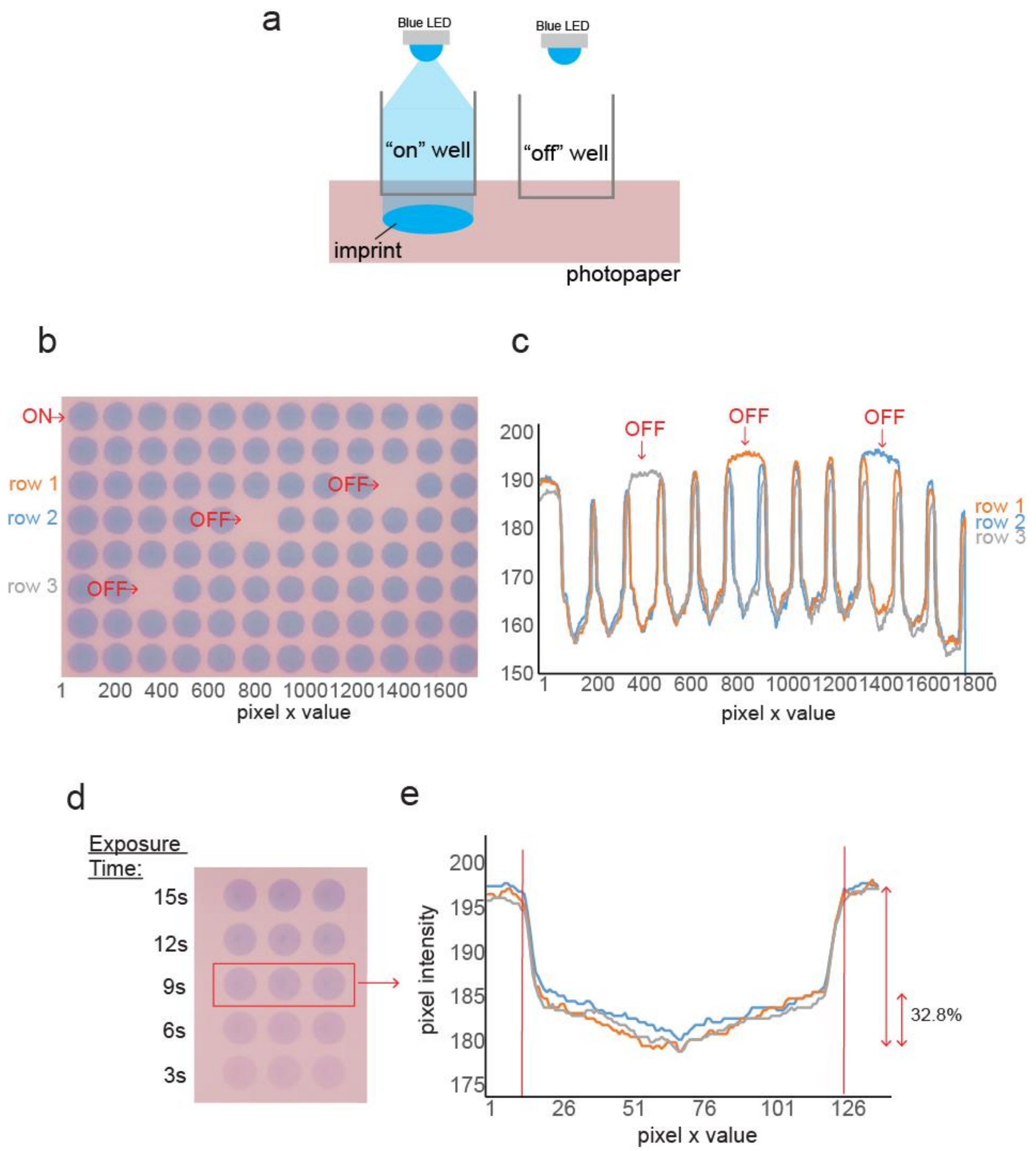

Supplementary Figure 1. Insulation of light between wells and homogeneity within wells. a Photopaper allows for imprinting and recording of light exposure underneath a 96 well plate from the blue LEDs[1]. **b** All wells of the optoPlate's blue LEDs were set to maximum except the 3 indicated as "OFF." The optoPlate was mounted on a 96 well plate with photopaper placed below the plate. No imprint was observed in OFF wells, despite all surrounding wells being set to maximum intensity for 5 minutes. This indicates that light spread between wells is negligible. **c** Quantification of the intensity across each row of wells of the exposed photopaper in **(b)** shows that OFF wells do not display detectable imprints from light exposure despite neighboring wells being set to maximum intensity. Data represents a line profile of the pixel intensity across the center of each row of wells. **d** Photopaper can also be used to analyze light uniformity within wells. Triplicate wells were exposed to blue light for varying times in order to find a non-saturating exposure time. 9s was chosen for quantification. **e** Line profile of the pixel intensities across the center of each well from the 9s exposure condition from **(d)**. Blue light intensity varies by 32.8% from the center to the edge of the wells (indicated with red lines). However, this non-uniformity is can be overcome either by 1) stimulating with strong blue light such that even the weaker light at the periphery is saturating for the optogenetic protein, or 2) shaking the cultures during growth, homogenizing the effective light intensity that the bacteria experience over time.

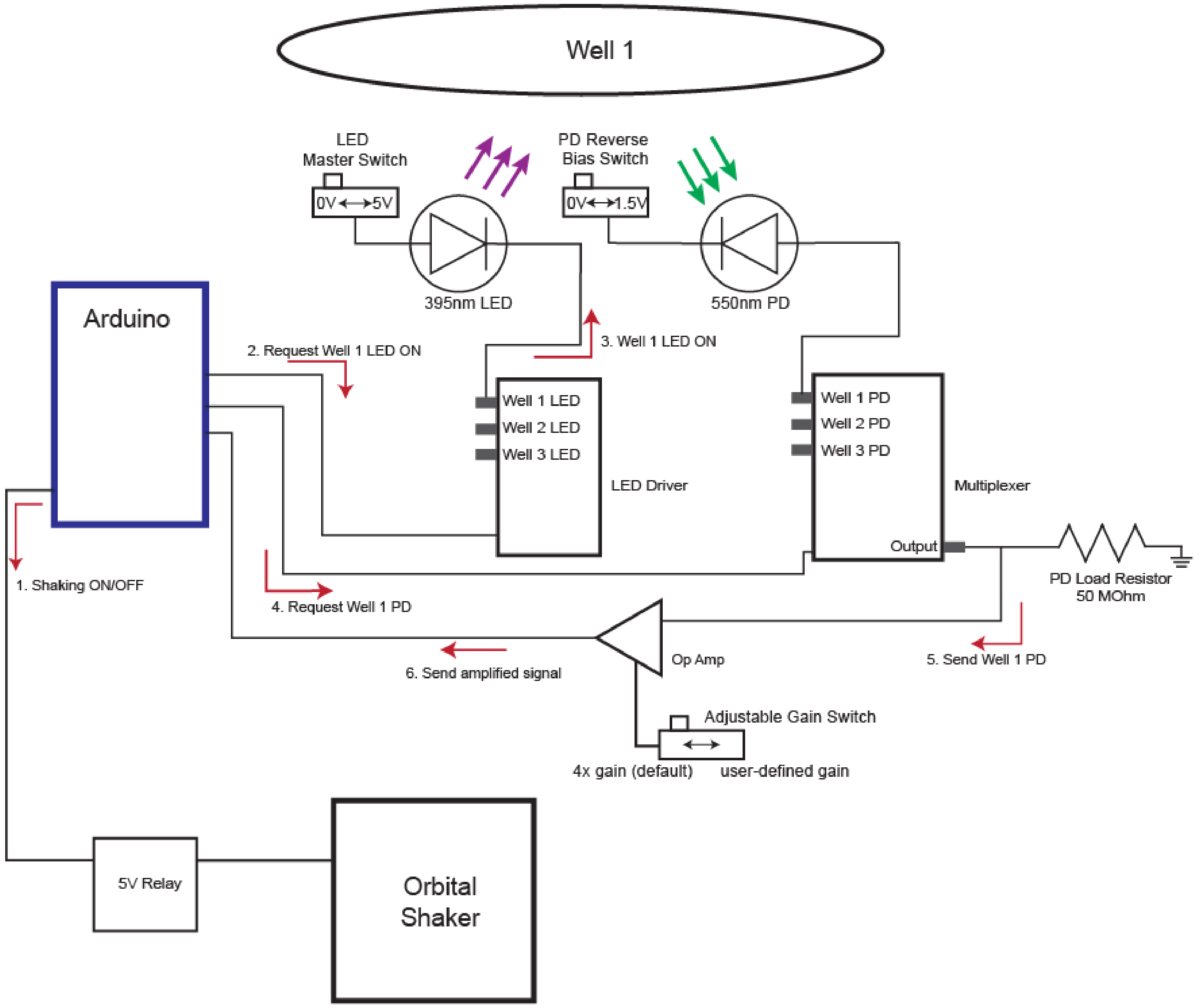

Supplementary Figure 2. oPR circuit diagram for fluorescence readings in a well. To acquire fluorescence readings for a well, the following sequence of events occurs: 1) Shaking is paused during measurements. 2) The oPR Arduino sends a signal to the LED drivers requesting that the UV LED for that well be switched on; 3) The LED driver switches on the LED for that well while leaving all other LEDs off. The LED 5V switch must be set to the 5V position for LEDs to turn on. This switch ensures that the LEDS are only operational when desired to prevent inadvertent illumination on samples or investigators. 4) The Arduino then sends a command to the multiplexer to select the photodiode (PD) for that well. The PD signal can be altered by changing the Reverse Bias switch. 0V (used in this study) allows detection of lower intensity signals by reducing the background signal produced by the photodiodes. 1.5V allows for detection of higher intensity signals by raising the point of signal saturation. This is because a greater maximum voltage can be produced by the photodiode than is possible without a reverse bias. However, this also causes a higher background signal when no fluorescence is present. 5) The signal from the PD is then sent to an amplifier that multiplies the signal by an adjustable gain. The Adjustable Gain switch can be set to 4x (used in this study) or a user defined gain (by adding a resistor of a desired value as labeled in the component diagram). 6) The final signal is sent to the Arduino for processing. A similar sequence of events takes place for OD readings, with additional communication with the optoPlate to coordinate illumination of the OD LED.

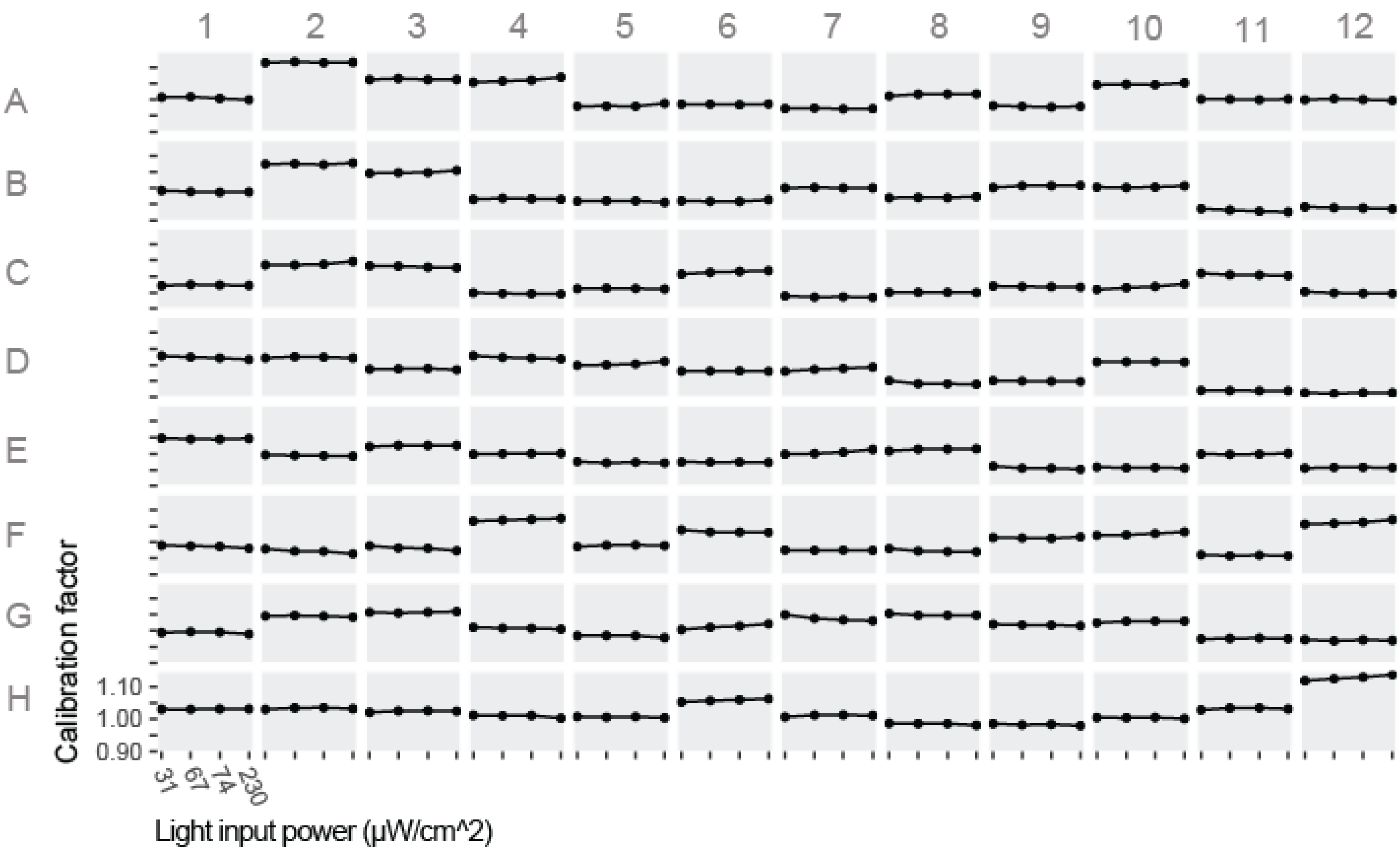

Supplementary Figure 3. Photodiode calibration factors are constant at different light input intensities. The calibration factor for each input intensity was calculated separately for each well at 4 different light intensities. In general, calibration factors were independent of input light power.

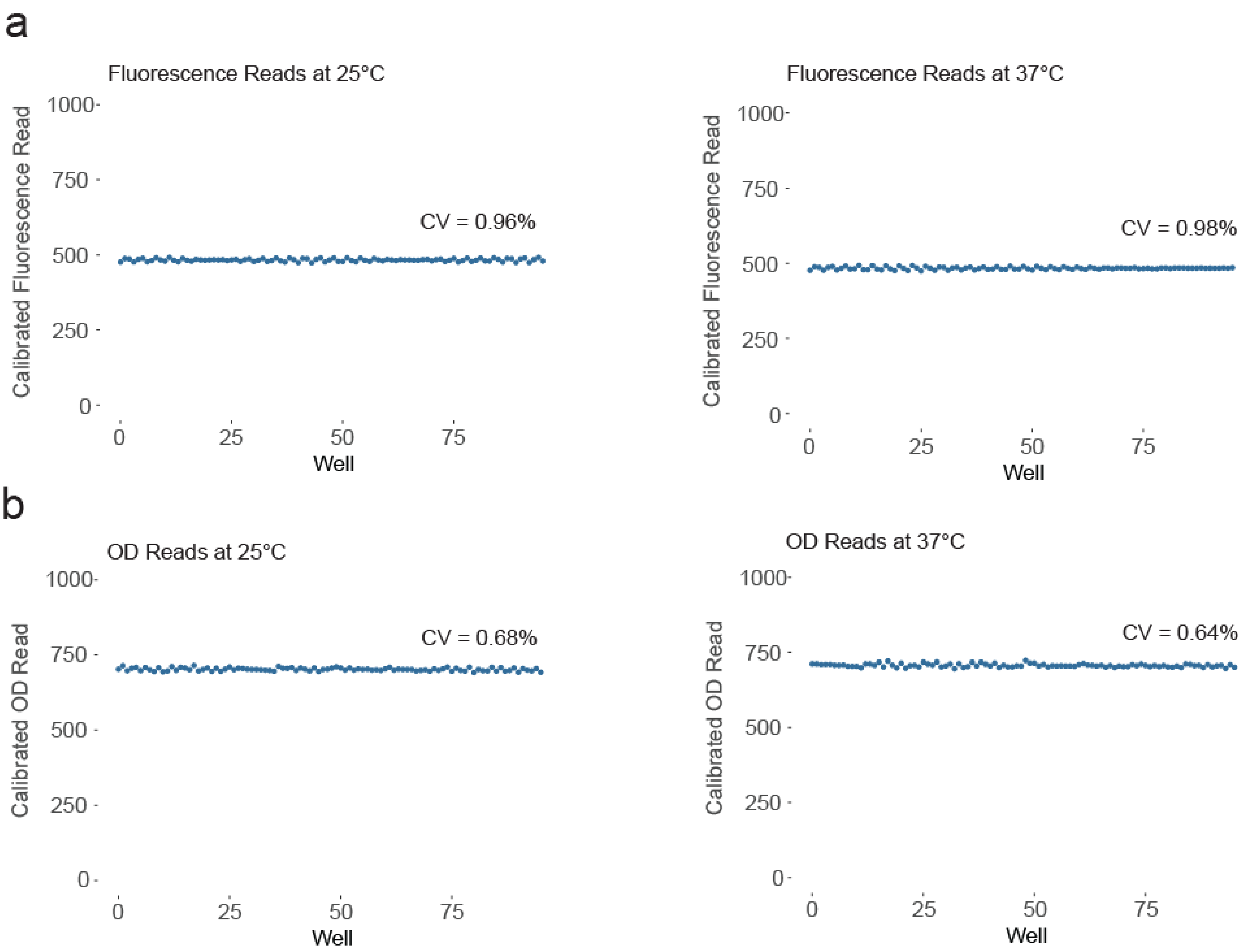

Supplementary Figure 4. Calibrated measurements do not vary as a function of temperature. a Calibrated fluorescence measurements were taken at 25°C using 40 µg/mL of lucifer yellow per well inside of a cell culture incubator. The cell culture incubator was then allowed to equilibrate at 37°C and measurements were repeated using the same calibration values. There was no significant change in measurement amplitude or variability of measurements between the two temperatures. **b** The same process was repeated for OD LED intensity with wells containing LB and again no significant temperature-dependent differences were observed.

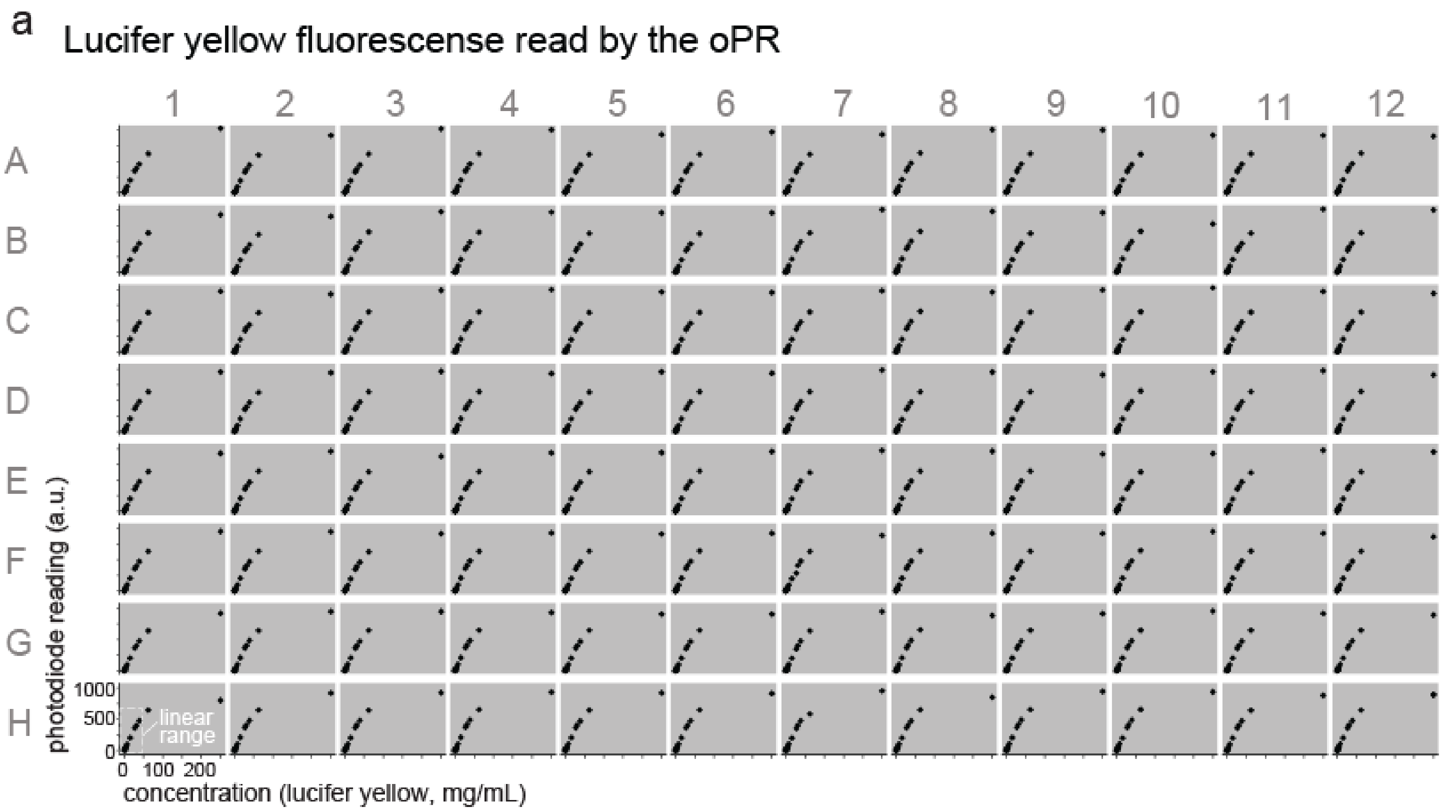

Supplementary Figure 5. Full range of photodiode fluorescence characterization
Fluorescence measurements of lucifer yellow dye were taken over a range of concentrations (2-250 µg/mL) in each of 96 wells in a calibrated oPR. Concentrations from 2-40 µg/mL are reproduced from **Figure 4**.

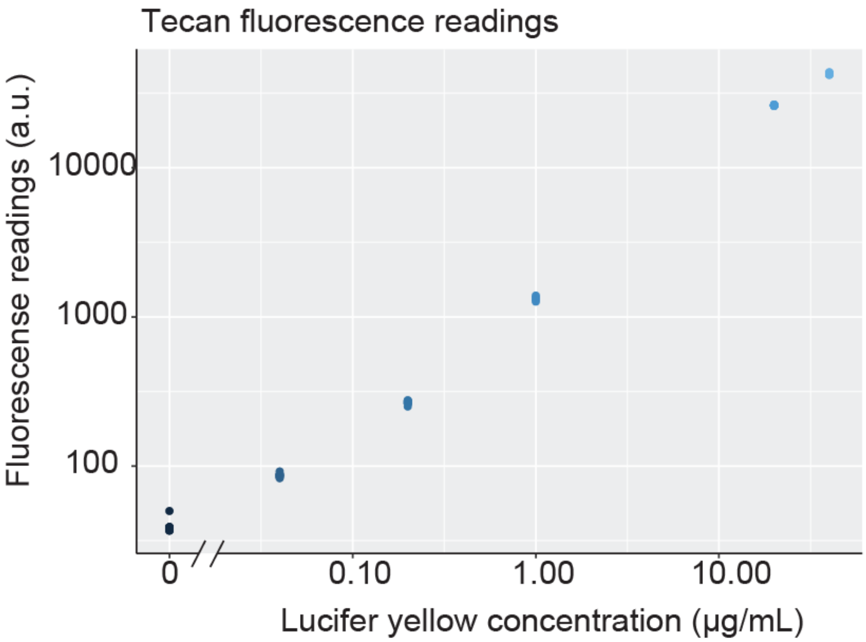

Supplementary Figure 6. Tecan limit of detection experiments. Lucifer yellow was diluted to several concentrations and measured using the Tecan Infinite M200. Based on this data, the limit of detection was calculated to be ~10 ng/mL. Each point represents the read from 1 well of lucifer yellow at the specified concentration.

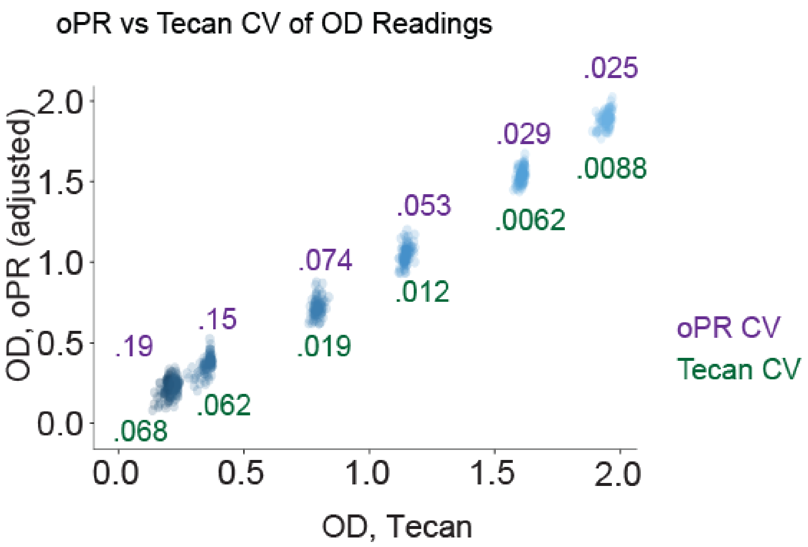

Supplementary Figure 7. Coefficient of variation for oPR vs Tecan OD readings. CV values were calculated for data shown in **Figure B**. CVs displayed above data points correspond to oPR measurements (magenta) at the corresponding concentration. CVs displayed below data points correspond to Tecan measurements (green).

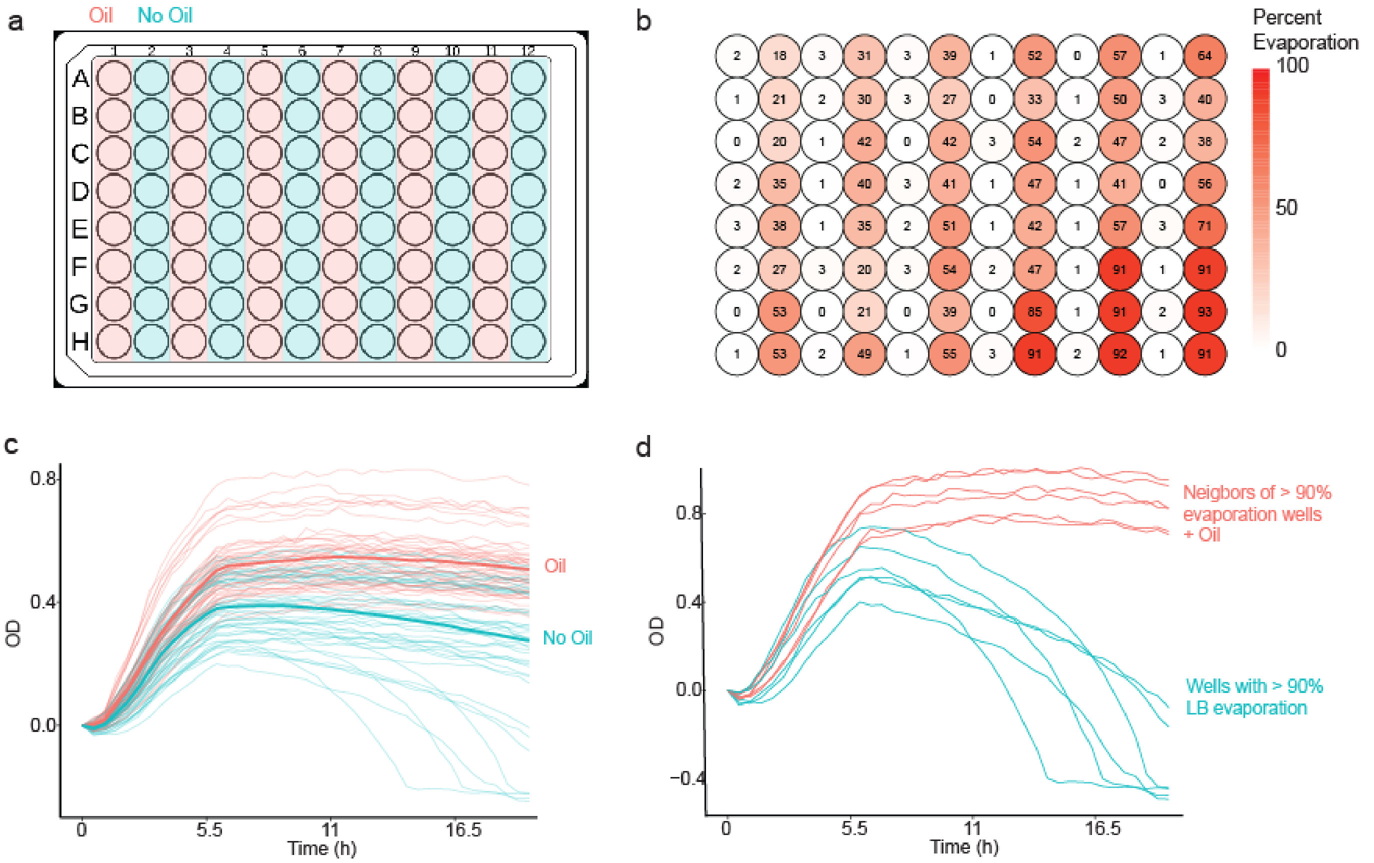

Supplementary Figure 8. Paraffin Oil prevents evaporation. a Bacteria were cultured in a 96-well plate with and without a 45 µL layer of paraffin oil for 18 hours of culture and measurement on an oPR with shaking at 1000rpm. **b** Percent LB evaporation from each well after the experiment described in (**a**). **c** OD readings over time from the experiment described in (**a**). Wells without paraffin oil exhibit an initial increase but subsequent decrease in OD readings, likely due to the fact that evaporation caused settling of dry residue on the outer edges of the well, creating an optically clear path through the center of the well, allowing greater light transmission than the initial liquid LB. By contrast, wells with paraffin oil exhibit a monotonic increase and plateau at ~ 6 hrs. **d** Examination of wells with most extreme evaporation compared to their neighbors with paraffin oil. Wells with >90% evaporation show a major decrease in OD readings over time (blue) while their direct oil-coated neighbors showed normal growth (red).

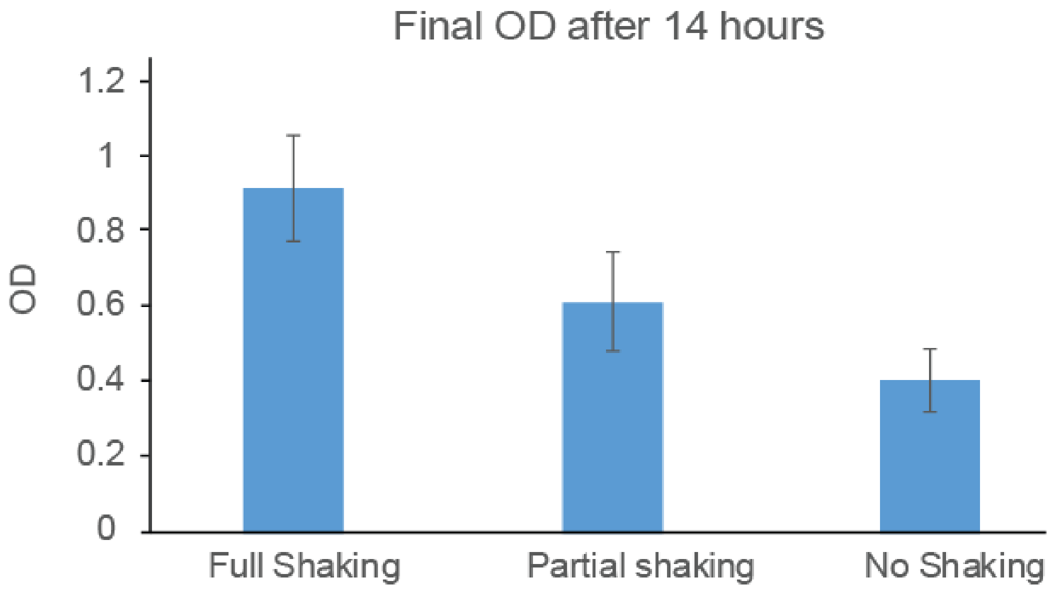

Supplementary Figure 9. Shaking effect on culture OD. Bacterial cultures were grown in 96 well plates for 14 hours with either constant shaking, 2 minutes of shaking every half hour, or no shaking. The final OD was measured, showing that increased shaking frequency led to higher final ODs. Each bar represents the mean of 96 wells with error bars representing 1 s.d.

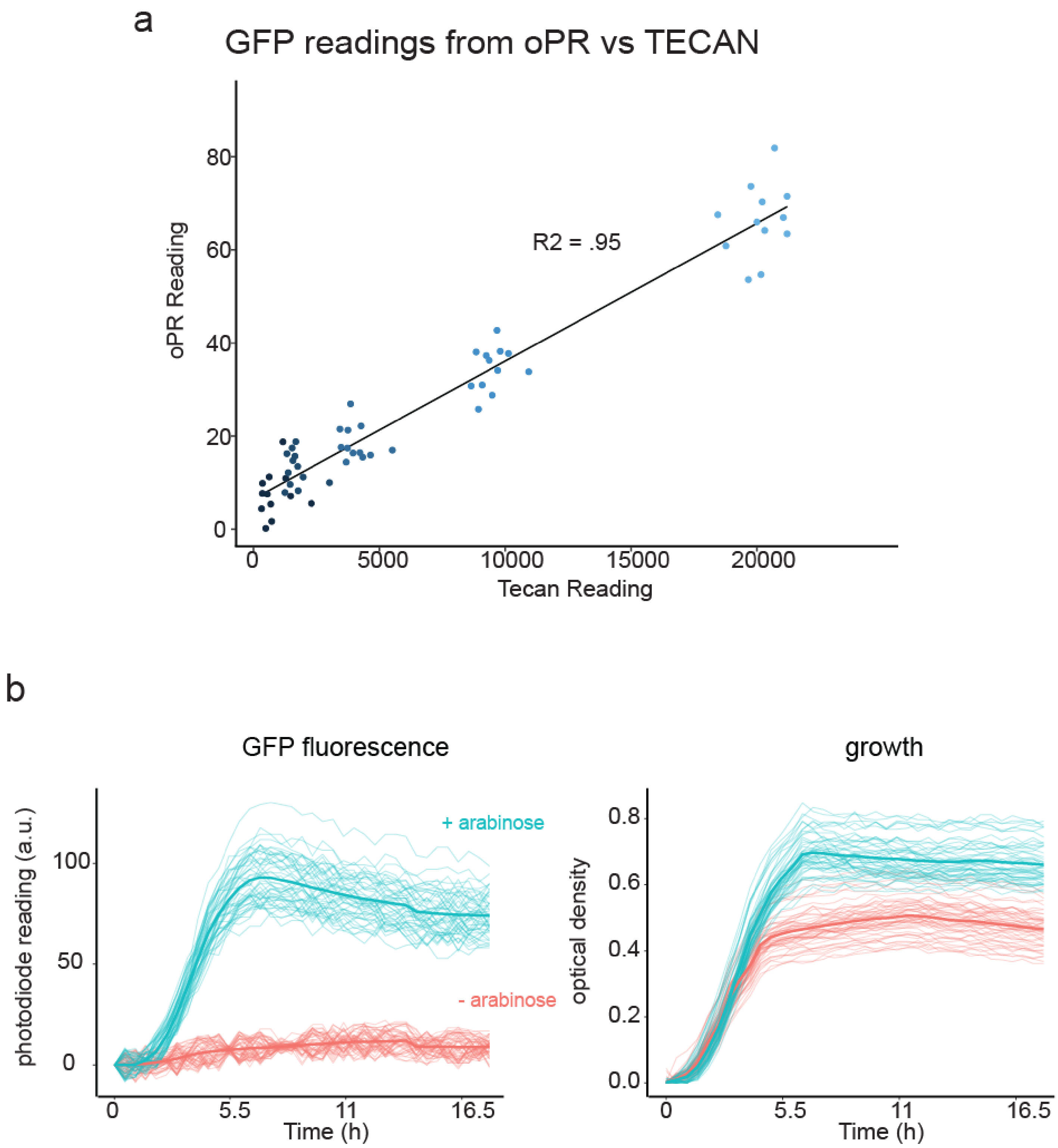

Supplementary Figure 10. oPR detection of GFP in live cultures. a Bacteria transformed with an arabinose inducible GFP expression plasmid were grown to saturation in 2 mL of LB + arabinose and diluted in a series of 2x dilutions. Each dilution was seeded in one row of a 96 well plate, and GFP intensities were read with both an oPR and Tecan. The oPR was able to detect GFP using a 475 nm excitation LED, and increases in fluorescence were linear with increases in fluorescence as measured by a Tecan plate reader. **b** Arabinose inducible GFP cultures were grown with or without arabinose for 18 hours, demonstrating the ability of the oPR to detect GFP expression during live time course experiments.

References

1. Höhener, T. C. *et al.* LITOS: a versatile LED illumination tool for optogenetic stimulation. *Sci. Rep.* **12**, 1–11 (2022).
